# Case report: VEXAS as an example of autoinflammatory syndrome in pulmonology clinical practice

**DOI:** 10.3389/fmed.2024.1340888

**Published:** 2024-01-26

**Authors:** Ewa Więsik-Szewczyk, Arkadiusz Zegadło, Agnieszka Sobczyńska-Tomaszewska, Marcelina Korzeniowska, Karina Jahnz-Rózyk

**Affiliations:** ^1^Department of Internal Medicine, Pneumonology, Allergology and Clinical Immunology, Central Clinical Hospital of the Ministry of National Defense, Military Institute of Medicine, National Health Institute, Warsaw, Poland; ^2^Department of Radiology, Central Clinical Hospital of the Ministry of National Defense, Military Institute of Medicine, National Health Institute, Warsaw, Poland; ^3^MedGen Medical Centre, Warsaw, Poland

**Keywords:** autoinflammation, mosaicism, *UBA1*, somatic mutations, respiratory manifestations

## Abstract

Lung involvement is not widely recognized as a complication of auto-inflammatory diseases. We present a broad approach to diagnose a severe form of autoinflammatory syndrome in an adult male patient. A 63-year-old Caucasian male presented with recurrent episodes of high fever, interstitial lung infiltration, and pleural effusion. Laboratory tests performed during the flares revealed lymphopenia and increased levels of C-reactive protein and ferritin. Broad diagnostic research on infections, connective tissue diseases, and malignancies yielded negative results. The patient’s symptoms promptly resolved upon the administration of glucocorticoids; however, they reappeared when the prednisone dose was reduced. All attempts to administer immunomodulatory and immunosuppressive medications were ineffective. During follow-up, autoinflammatory syndrome was suspected; however, no pathological variants of monogenic autoinflammatory diseases were identified by genome-exome sequencing. The patient did not respond to interleukin 1 blockade with anakinra. He died due to multi-organ failure, and his condition remained unresolved until the first reported description of vacuole, E1 enzyme, X-linked, autoinflammatory, and somatic syndrome (VEXAS). We describe the diagnostic traps and reasoning process involved in establishing that the patient’s symptoms were autoinflammatory in nature based on clinical symptoms, in addition to the proof of concept gained from genetic reevaluation and identification of pathogenic variants in the *UBA1* gene. The aim of this review is to increase the awareness of VEXAS among pulmonologists. Genetic screening for *UBA1* should be considered in patients with recurrent pneumonitis of unknown origin with elevated inflammatory markers and signs of cytopenia, especially if they require chronic steroids to control the disease. Respiratory manifestations are part of VEXAS; these may be dominant in the course of the disease and severe at presentation.

## 1 Introduction

Lung involvement is a well-recognized complication of autoimmune systemic diseases; there is an increasing awareness of its importance and significance in patient prognosis and survival (1). In contrast, autoinflammatory diseases, which are rare, are not widely recognized to be associated with lung involvement. Here, we present long path to the diagnosis and treatment of a severe form of autoinflammatory syndrome in an adult male patient. We describe the diagnostic traps and reasoning processes involved in the phases: first, the patient’s symptoms were autoinflammatory in nature based on clinical reasoning; and second, the proof of the concept gained from repeated genetic evaluation, which led to the diagnosis of vacuoles, E1 enzyme, X-linked, autoinflammatory, and somatic syndrome (VEXAS), as described by Beck and colleagues (2) after patient death.

## 2 Case description and diagnostic assessment

The patient was a 63-year-old Caucasian man with a history of episodes of a fever reaching up to 39.8^ο^C, with chills and weakness. Fever appeared without any prodromal symptoms, recurred regularly every 3 weeks, and lasted for 5–7 days from January 2014 to March 2014. The episode in April 2014 was accompanied by cough, dyspnea, and pneumonia, which resulted in admission to a local hospital. Upon admission, the patient had elevated C-reactive protein (CRP) levels, low-grade anemia, transient leukopenia, and mild thrombocytopenia. None of his symptoms improved after antibiotic treatment; however, they were promptly resolved when the patient was administered glucocorticoids (40 mg prednisone/day). Six months before these episodes, the patient had an episode of orchitis and was treated with ciprofloxacin. His family history of chronic diseases was unremarkable. He denied the use of tobacco, alcohol, or illicit drugs.

In May 2014, he was referred to our department for further evaluation. On admission, the patient was stable without fever and continued to receive 30 mg prednisone/day. Bronchoscopy revealed a normal bronchial tree. In the immunophenotyping of cells obtained from the bronchoalveolar lavage, the low percentage of lymphocytes was noteworthy; CD45+ lymphoid cells constituted only 0.3% of the white blood cells and 0.2% of CD3 + T lymphocytes, including CD4+ (30%) and CD8+ (5%). A blind lung biopsy revealed no specific changes. Plethysmography findings were within the reference range (June 2014). A detailed evaluation for chronic infections, systemic connective tissue diseases, and solid malignancies, including positron emission tomography scanning, were unremarkable. Bone marrow aspirate biopsy and histopathology revealed normal cellularity, which was age-appropriate. The presence of vacuoles has not been described. Owing to the stabilization of the patient’s condition, normalization of laboratory inflammatory markers, normal pulmonary function test results, and resolution of radiological abnormalities, we recommended the complete discontinuation of glucocorticoids.

**Figure 1:**
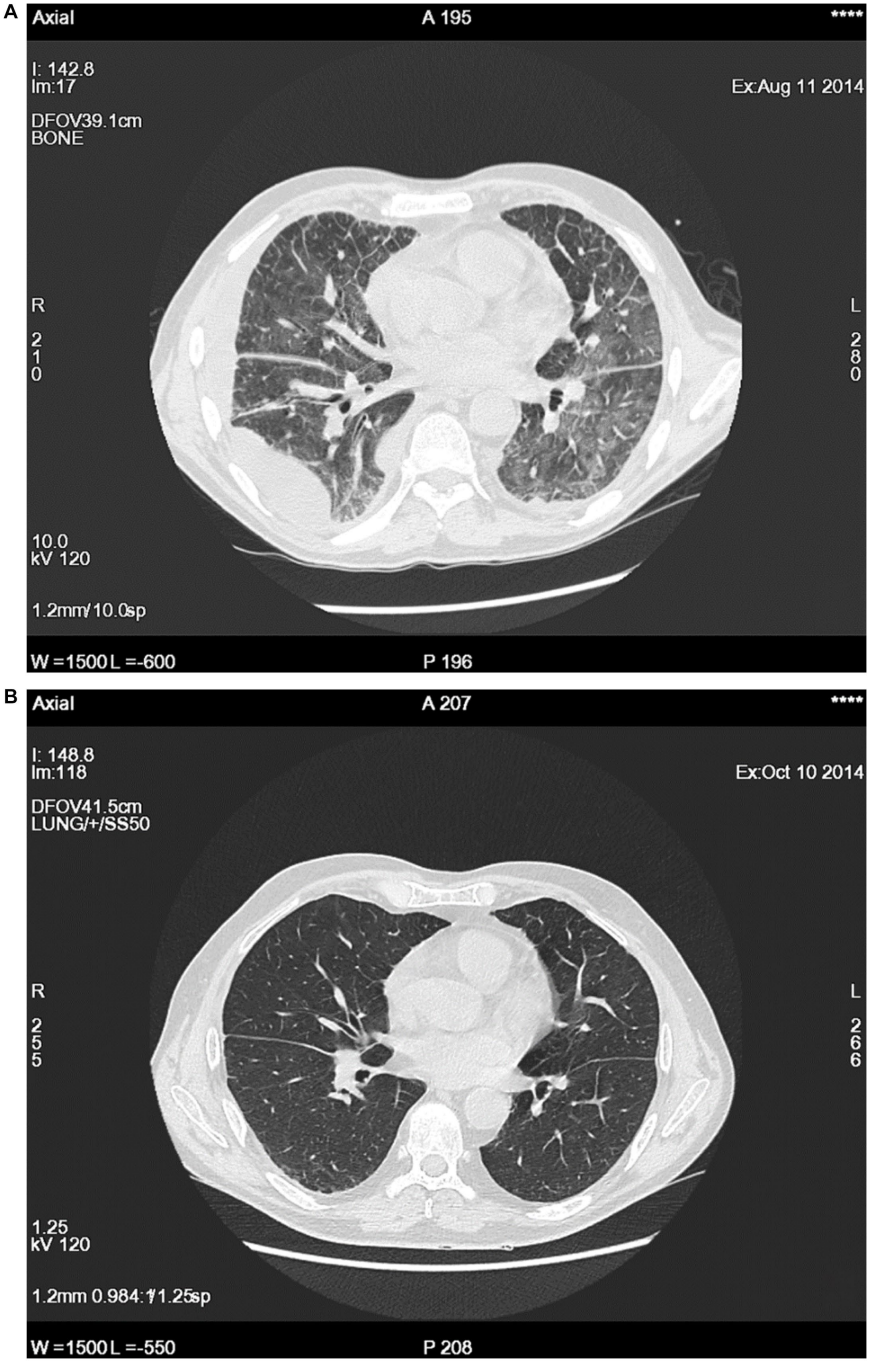
Examples of imagining studies during a flare up **(A)** and after 2 months of steroids treatment **(B)**.

However, in August 2014, when the prednisone dose was reduced to 15 mg/day, a high-grade fever with subsequent dyspnea reappeared, without any prodromal symptoms. On physical examination, the patient was febrile with signs and symptoms of pneumonia. Chest computed tomography (CT) revealed bilateral ground-glass opacities, inflammatory consolidation with bronchial wall thickening, reticulation, thickened septal lines in the parenchyma, subpleural micronodules, and effusion in the right pleural cavity (Figure 1A). The patient denied any additional symptoms, such as joint pain or swelling, muscle pain, skin rash, chondritis, polymyalgia rheumatic-like symptoms or lymphadenopathy. To control the disease, 40 mg of prednisolone was administered daily. Normalization of inflammatory markers and regression of radiological abnormalities (Figure 1B) were accompanied by a clinical improvement.

Despite chronic steroid therapy (15–20 mg/prednisolone per day), fever and dyspnea reappeared regularly every 3–4 weeks. The patient’s condition improved only after an increase in the steroid dose. Episodes of fever documented in the patient’s diary from 2015 to 2019 lasted, on average, 5 days, the shortest being 2 days and the longest 7 days, with a temperature range from 37.2 to 39.2°C. The patient was evaluated by multiple specialists. Repeat bronchoscopy and CT were inconclusive. Pulmonary function tests (PFTs) performed during the flare up revealed severe abnormalities (Table 1), and laboratory tests revealed lymphopenia and elevated levels of CRP and ferritin (Table 2). The lymphocyte subpopulation analysis showed a significantly lower proportion and number of B cells, with a high proportion of switched memory B cells and plasmablasts (Table 3). Among the T lymphocytes, a high proportion of Th17 was observed (Table 3).

**Table 1:** Results of body plethysmography and diffusion examination during the flare up.

	Pred	Pre	%(Pre/Pred)	*p*	Z-score
VC MAX	3.63	1.43	39	0.12	−4.00
FRCpleth	3.37	1.99	59	1.95	−2.30
RV	2.41	1.40	58	1.51	−2.46
TLC	6.10	2.83	46	0.04	−4.68
RV % TLC %	40.09	49.45	123	94.86	1.71
DLCO_SB mmol/(mln*kPa)	7.51	2.17	29	0.05	−5.71

**Table 2:** Examples of laboratory results obtained during different time points and clinical states.

	19 May 2015 4 days after flare up	08 Dec 2016 3 days after flare up	11 Jun 2019 day 4 of flare up, temp. 38.2^ο^C	24 Sep 2019 2 weeks asymptomatic	22 Oct 2019 day 1 of fever
Inflammatory markers					
ESR (0–20 mm/h)	NR	109	NR	16	NA
CRP (0–0.8 mg/dL)	12	12.3	23.2	0.2	44.6
Ferritin (30–400 ng/mL)	NR	NR	NR	1710	2,770
Complete blood count					
WBCx10^9^/l (4.0–10.0)	4.40	7.53	5.57	2.82	6.77
RBCx10^12^/l (3.5–5.5)	2.84	3.49	3.44	3.68	4.02
Hgb g/dl (11.0–18.0)	9.0	11.5	11.4	12.4	14.3
HCT % (35–55)	29	36	34	37	43
MCV fL (80–100)	102	103	90	101	107
MCHC g/dl (31–37)	31	31.9	33.5	33.3	33.3
PLT x 10^9^/l (150–400)	223	158	90	128	97
Lymph x10^3^/uL (0.9–4.5)	0.84	0.84	0.26	0.69	0.34
NEUT x10^3^/uL (1.9–8.0)	3.43	6.30	5.06	2.02	6.16
Mono x10^3^/uL (0.16–1.0)	0.12	0.35	0.24	0.10	0.15

WBC, white blood cell count; Hgb, hemoglobin; HCT, hematocrit; PLT, platelet; CRP, C-reactive protein; ESR, erythrocyte sedimentation rate; NR, not reported.

**Table 3:** Lymphocyte subset analysis performed during the flare up.

Cells	% Total	Lymphocytic field (reference values)			
T cells (CD3+)	20.1	85.4	(60–82%)	569	(900–2,100 c/ul)
Th cells (CD3 + CD4+)	11.5	48.8	(33–55%)	323	(500–1,300 c/ul)
Ts cells (CD3 + CD8+)	8.4	35.6	(18–40%)	238	(280–900 c/ul)
CD4:CD8		1.4	1.1–2.8		
NK cells (CD3 + CD16 + CD56+)	2.8	11.8	(7–23%)	79	(90–630 c/ul)
B cells (CD19+)	0.7	2.8	(5–16%)	20	(120–400 c/ul)
B cells				% CD19 + (reference %)	
Transitional B cells IgM++IgD++CD38++CD27-CD21+				1.4	(0.9–6.3)
Naïve B cells IgM + IgD++CD38 + CD27-CD21+				18.0	(53.3–86.0)
Non-switched memory B cells (MZ-like B cells) IgM++IgD + CD38 + CD27 + CD21+				4.3	(3.3–12.8)
Class-switched memory B cells IgM-IgD-CD38 + CD27 + CD21+				4.3	(4.0–22.1)
CD21^low^ B cells IgM + IgD + CD38^low^CD27-CD21^low^				54.7	(0.6–7.6)
Plasmablasts IgM−/+IgD-CD38+++CD27++CD21+				19.8	(0.1–1.5)

T cells	%CD4+ (reference %)		%CD8+ (reference %)	
Naïve T cells CD45RA + CD197+	22.5	18.2–53.2	2.2	18.6–70.6
Effector T cells CD45RA+ CD197-	1.4	1.1–9.0	65.0	4.0–24.0
Central memory T cells CD45RO + CD197+	48.2	8.3–27.1	7.1	0.5–5.4
Effector memory T cells CD45RO + CD197-	27.9	17.2–51.4	25.7	16.7–42.6
Th17 CD45RO + CD196+	55.2	10.8–39.0	–	–
Recent thymic emigrants T cells CD45RA + CD62L + CD31+	3.5	14.4–38.3	3.1	16.7–64.7

In August 2015, immunomodulatory and immunosuppressive treatments were initiated as steroid-sparing agents. The patient initially received azathioprine (150 mg/day), mycophenolate mofetil (2 × 1000 mg/day), and hydroxychloroquine (200 mg/day). All therapeutic attempts, except for steroids, were ineffective.

In 2019, autoinflammatory syndrome was suspected. During the flare up, the interleukin 1 (IL-1) inhibitor anakinra was administered, initially one dose of 100 mg subcutaneously (sc), followed by one 200 mg sc dose, as a new therapeutic trial. However, no significant clinical effects were observed. In November 2019, whole exome sequencing (without copy number variation analysis) was performed to identify known pathogenic and likely pathogenic variants described in the ClinVar database with the detailed analysis of variants (pathogenic/likely pathogenic/variation of uncertain significance (VUS)) in 578 genes associated with autoinflammatory diseases and inborn errors of immunity. The analysis did not reveal any pathogenic variants; only a few VUS-type variants were identified and described for clinical analysis. However, they did not support the diagnosis of autoinflammatory syndrome. Ultimately, the patient received cyclophosphamide (CYC) infusion, which was complicated by severe infection. The patient died of multi-organ failure as an unresolved case in May 2020. In October 2020, following the first description of VEXAS, genetic reevaluation was performed.

Pathogenic variants of the *UBA1* gene: NM_003334.3:c.121A > G, NP_003325.2:p. Met41Val was detected in about 65% of the cells (Supplementary Figure S1). This variant is classified as pathogenic in ClinVar database and is associated with VEXAS syndrome. The identification of pathogenic somatic mutations in *UBA1* led to the correct diagnosis on November 4, 2020.

## 3 Discussion

We report a case of recurrent fever with pneumonia that was steroid-dependent and resistant to immunosuppressive treatment. The differential diagnosis of autoinflammatory syndromes in adults is challenging. At the first instance, infections, malignancies, and systemic autoimmune diseases were excluded. We performed serological, imaging, and pathological evaluations that did not explain the patient’s condition; therefore, we suspected autoinflammatory syndrome, which was mainly based on the clinical pattern of recurrent inflammatory syndromes accompanied by high levels of inflammatory markers. The treatment trial with IL-1 inhibition, which, if effective, would have supported the suspicion autoinflammatory syndrome, was negative. The same result was observed during the initial genetic evaluation. However, the results were misleading. Despite comprehensive investigations, the diagnosis was unknown until the first reported description of VEXAS syndrome. VEXAS is an adult-onset autoinflammatory syndrome due to somatic mutations affecting *UBA1* reported by Beck and colleagues (2). The first patient, as in our report, was identified among unresolved cases with unexplained inflammation. Thus, there is an argument to re-evaluate similar cases in pneumonology practice. Although the frequency of this condition is unknown, it appears to be the most common autoinflammatory syndrome in adults. The prevalence estimates are approximately 1 in 4269 males aged 50 years and 1 in 26,238 females aged 50 years (3). Currently, in males aged >50 years with an inflammation of unknown origin, VEXAS should be included early in the differential diagnosis. However, there are no available recommendations regarding when and for whom genetic evaluation should be performed. Recently, Maeda et al. (4) proposed a scoring system that can efficiently identify patients with *UBA1* variants. Their clinical scoring system included age > 50 years, cutaneous lesions, lung involvement, chondritis, and macrocytic anemia; it predicts *UBA1*-positive patients as those with a maximum score of 6 and *UBA1*-negative patients as those with low scores of 0–2, while recommending that patients with intermediate scores of 3–5 definitely undergo *UBA1* testing (4). The retrospectively presented patient had positive scores regarding age and pulmonary involvement. In the present analysis of this case, the hematologist excluded central anemiaand diagnosed anemia related to chronic disease, which in the retrospective analysis, seemed to be questionable and should be re-evaluated, resulting in a score of 4 points. In another study, a higher initial mean corpuscular volume predicted the diagnosis of VEXAS in a rheumatology cohort (5). It is important to closely monitor hematologic abnormalities in cases of suspected VEXAS, as they can be absent at the initial phase or can fluctuate, as in the present patient.

VEXAS is multidisciplinary and involves numerous branches of internal medicine, including pulmonology. Currently, VEXAS is recognized in patients diagnosed with myelodysplastic syndrome and various rheumatological and dermatological conditions (5–13). The present case is unique, as fever and lung involvement dominated the clinical picture, without dermatosis, arthritis, recurrent chondritis, polymyalgia rheumatica-like symptoms or bone marrow abnormalities, which are often present in VEXAS. Reduced awareness of the disease among pulmonologists may result in omission of the diagnosis of VEXAS in patients with predominant pulmonary involvement. The pulmonological aspects of VEXAS are less precisely characterized, although they are reported as constant clinical features during follow-up and seldom as the main clinical complaint (14). In 2022, the first systematic review aimed to summarize the respiratory manifestations of VEXAS as described in the literature (15). The most commonly described manifestation was pulmonary infiltrates, present in 43% of patients, often coexisting with other lung pathologies, such as nonspecific interstitial pneumonia, pulmonary vasculitis, and pleural effusion. In our patient, several abnormalities were present on CT, including an abnormal PFT during the flare up. In a cohort study conducted on all patients with VEXAS syndrome evaluated at the Mayo Clinic (16) the authors reported respiratory symptoms in 93% of the patients, accompanied by skin lesions and fever in 91 and 82% of the patients, respectively. Chest CT showed abnormalities in 91% of patients; however, these were nonspecific. PFTs were available for a minority of patients (40%) who presented with mild restrictive impairment or normal results. In general, the authors concluded that the pulmonary manifestations were relatively nonspecific. In a French cohort, lung pathology was one of the most common clinical features of VEXAS and was associated with mortality (12). When assessing phenotype–genotype correlations, lung infiltrates were more common in those with *UBA1* p.Met41Thr or p.Met41Val mutations than in those with p.Met41Leu (15), which also occurred in the present case. The currently available data on lung involvement may be biased because the reported cohorts were recruited mainly by rheumatologists or hematologists. Therefore, further studies including pulmonology cohorts are required.

The therapeutic approach to VEXAS is challenging, and data are limited and inhomogeneous (7, 15). Steroids are often the first choice and administered in doses ≥20 mg once daily; this is the only therapeutic approach to improve the inflammatory manifestations (7). Among anti-inflammatory drugs administered to decrease the corticosteroid dose, partial or negative responses are often observed (7). From the onset of the disease, the patient required >20 mg/day of prednisolone to control inflammation, and none of the immunosuppressive or immunomodulatory steroid-sparing agents provided additional benefits. Based on the assumption of an autoinflammatory condition in which excessive production of inflammatory cytokines occurs, anakinra was used, although without any effect. According to a recent systematic review, anti-IL-1-directed therapy was used in 6.0% of reported patients (17). Five patients received anakinra and two received canakinumab. Interestingly, two patients treated with anakinra and one patient treated with canakinumab received combination therapy with cyclosporin A. (17). Blockade of IL-6 with tocilizumab is another anti-inflammatory approach (18). In one patient with VEXAS and a previous diagnosis of spondyloarthropathy, treatment with intravenous immunoglobulin and an IL-17 inhibitor was effective (19). Furthermore, Th17 lymphocytes were present at a very high proportion in our patient, which supports this idea. Currently, data are accumulating regarding the efficacy of Janus kinase inhibitors, azacitidine, or allogeneic stem cell transplantation; however, treatment should be individualized based on the endotype of the disease, comorbidities, and the overall patient condition (16, 20–22). Therefore, there is an urgent need to collect clinical data over longer follow-up periods (23).

The present case report provides insights into the 7-year follow-up of an adult patient with autoinflammatory syndrome and predominant lung involvement. Clinical management and decision-making were more difficult when the patient’s condition was not recognized in the medical literature prior to death. A limitation of our description is that data from the patient’s first hospitalization in a local hospital were not available. Follow-up from the first admission to our institution is presented as precisely as possible. Our aim was to increase awareness of VEXAS among pulmonologists. As the primary take-away from this case report, we would like to propose that genetic screening for *UBA1* should be considered in patients with recurrent pneumonitis of unknown origin with elevated inflammatory markers and signs of cytopenia, especially if they require chronic steroids to control the disease. Based on the present case, respiratory manifestations are part of the primary disease and may be severe.

## Data availability statement

The original contributions presented in the study are included in the article/Supplementary material, further inquiries can be directed to the corresponding author.

## Ethics statement

Ethical approval was not required for the studies involving humans because the studies were conducted in accordance with the local legislation and institutional requirement. The participant provided his written informed consent to diagnostic and therapeutic procedures. Written informed consent was obtained from the individual(s) for the publication of any potentially identifiable images or data included in this article.

## Author contributions

EW-S: Conceptualization, Data curation, Formal analysis, Investigation, Methodology, Supervision, Writing – original draft, Writing – review & editing. AZ: Data curation, Visualization, Writing – review & editing. AS-T: Data curation, Visualization, Writing – review & editing. MK: Investigation, Writing – review & editing. KJ-R: Supervision, Writing – review & editing.
