# Supplementary material for: Case report: VEXAS as an example of autoinflammatory syndrome in pulmonology clinical practice
Source: Front Med (Lausanne). 2024 Jan 26;11:1340888. doi: 10.3389/fmed.2024.1340888 (PMC10858452; doi:10.3389/fmed.2024.1340888)
Supplement: Supplementary file 1 [file Data_Sheet_1.docx]

B


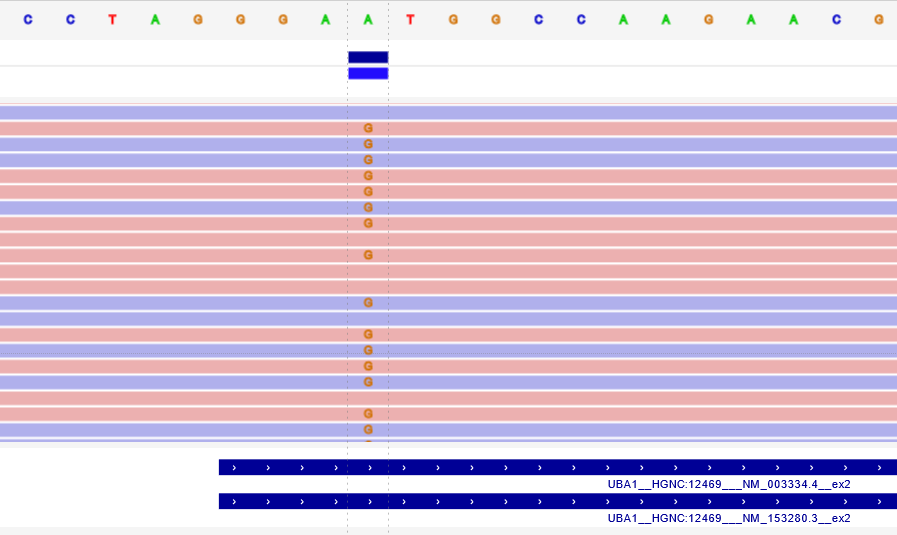

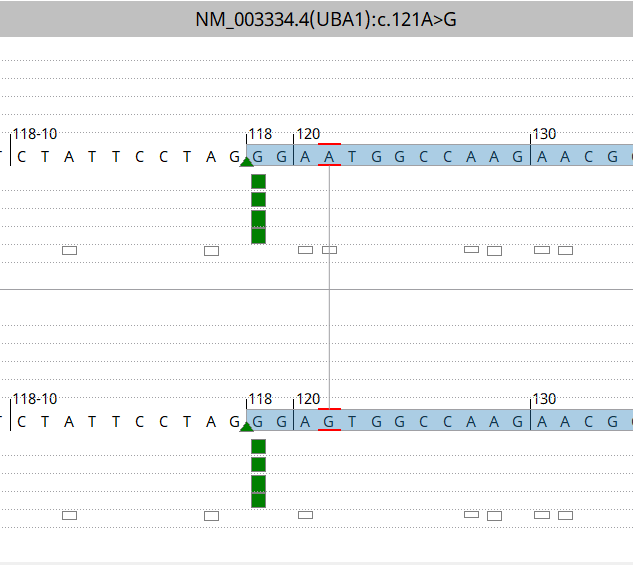

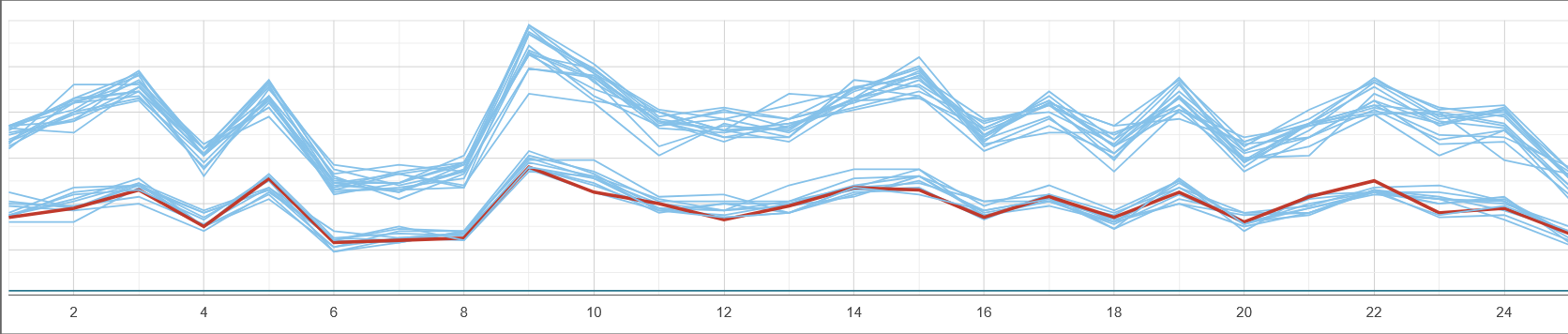

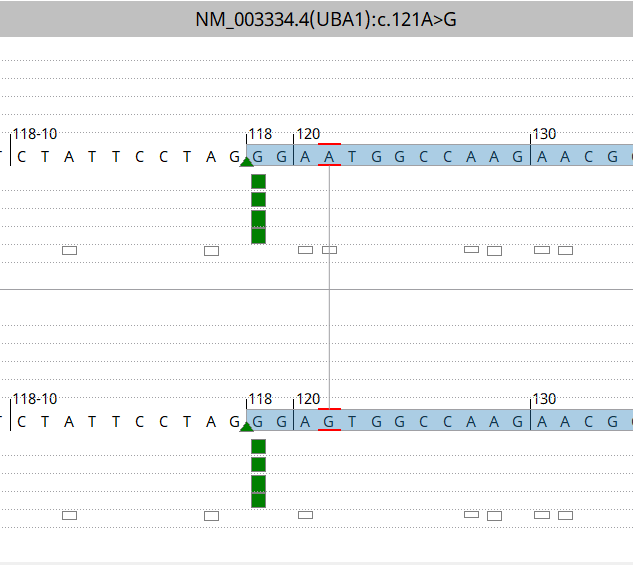


C

A

C

B


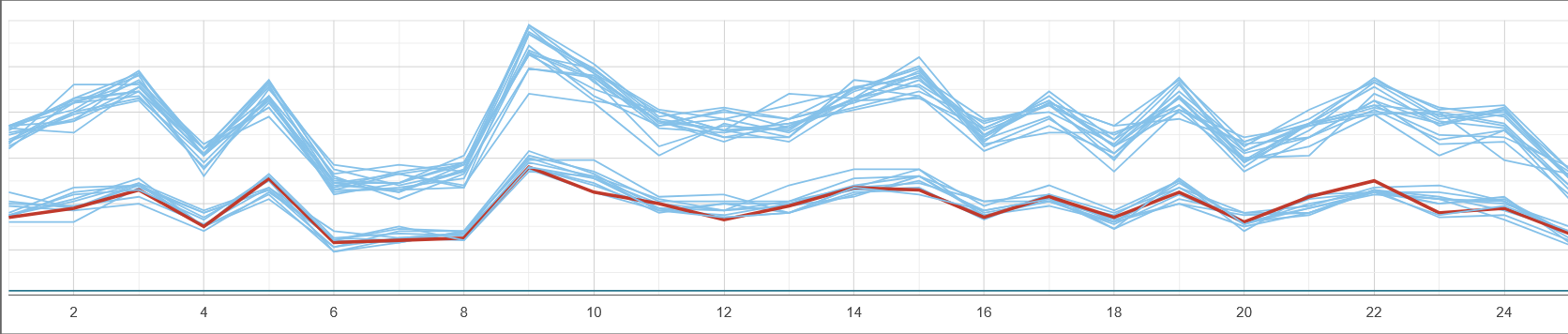


Fig 1S. Identification of a pathogenic variant p.Met41Val (c.121A>G) in exon 2 of *UBA1* gene during WES analysis (A). According to splice prediction, the missens variant does not affect the native splice site (B). No copy number variation variants were detected in the UBA1 gene (C).
